# Rapid discrimination of *Shigella* spp. and *Escherichia coli via* label-free surface enhanced Raman spectroscopy coupled with machine learning algorithms

**DOI:** 10.3389/fmicb.2023.1101357

**Published:** 2023-03-08

**Authors:** Wei Liu, Jia-Wei Tang, Jing-Yi Mou, Jing-Wen Lyu, Yu-Wei Di, Ya-Long Liao, Yan-Fei Luo, Zheng-Kang Li, Xiang Wu, Liang Wang

**Affiliations:** ^1^School of Medical Informatics and Engineering, Xuzhou Medical University, Xuzhou, Jiangsu, China; ^2^Laboratory Medicine, Guangdong Provincial People’s Hospital, Guangdong Academy of Medical Sciences, Southern Medical University, Guangzhou, Guangdong, China; ^3^The First School of Clinical Medicine, Xuzhou Medical University, Xuzhou, Jiangsu, China

**Keywords:** diarrheal disease, *Shigella* spp., *Escherichia coli*, machine learning, deep learning, Raman effect, SERS spectra

## Abstract

*Shigella* and enterotoxigenic *Escherichia coli* (ETEC) are major bacterial pathogens of diarrheal disease that is the second leading cause of childhood mortality globally. Currently, it is well known that *Shigella* spp., and *E*. *coli* are very closely related with many common characteristics. Evolutionarily speaking, *Shigella* spp., are positioned within the phylogenetic tree of *E*. *coli*. Therefore, discrimination of *Shigella* spp., from *E*. *coli* is very difficult. Many methods have been developed with the aim of differentiating the two species, which include but not limited to biochemical tests, nucleic acids amplification, and mass spectrometry, etc. However, these methods suffer from high false positive rates and complicated operation procedures, which requires the development of novel methods for accurate and rapid identification of *Shigella* spp., and *E*. *coli*. As a low-cost and non-invasive method, surface enhanced Raman spectroscopy (SERS) is currently under intensive study for its diagnostic potential in bacterial pathogens, which is worthy of further investigation for its application in bacterial discrimination. In this study, we focused on clinically isolated *E*. *coli* strains and *Shigella* species (spp.), that is, *S*. *dysenteriae*, *S*. *boydii*, *S*. *flexneri*, and *S*. *sonnei*, based on which SERS spectra were generated and characteristic peaks for *Shigella* spp., and *E*. *coli* were identified, revealing unique molecular components in the two bacterial groups. Further comparative analysis of machine learning algorithms showed that, the Convolutional Neural Network (CNN) achieved the best performance and robustness in bacterial discrimination capacity when compared with Random Forest (RF) and Support Vector Machine (SVM) algorithms. Taken together, this study confirmed that SERS paired with machine learning could achieve high accuracy in discriminating *Shigella* spp., from *E*. *coli*, which facilitated its application potential for diarrheal prevention and control in clinical settings.

**Figure:**
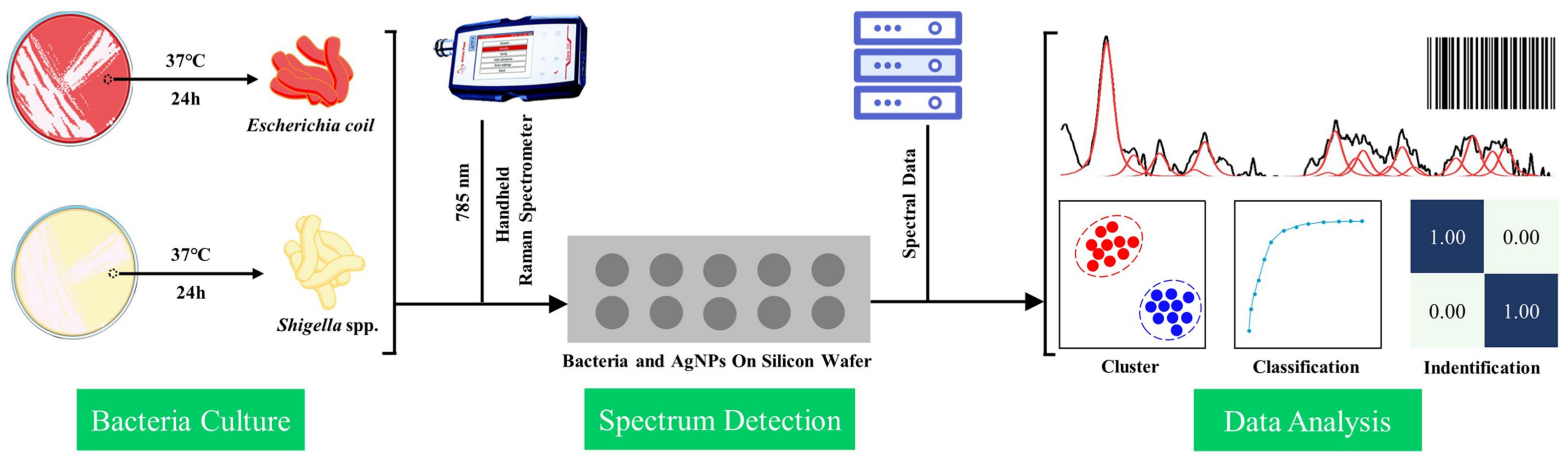
Graphical abstract

Graphical abstract

## Novelty statement

In this study, we achieved the rapid and accurate predication of *Shigella* spp., and *E*. *coli* with high accuracy by using surface enhanced Raman spectroscopy paired with machine learning algorithms, among which convolutional neural network showed the best performance. In addition, DeconvNet method was used for generating 2-dimensional unique barcodes for *Shigella* spp., and *E*. *coli*, which facilitated the rapid recognition of the two species.

## Highlights

*-Shigella* spp., and *E*. *coli* SERS spectra had unique characteristic peaks.
*-Shigella* spp., could be discriminated from *E*. *coli* with high accuracy *via* SERS.
-Deconvolution generated unique SERS barcodes for *Shigella* spp., and *E*. *coli*.

## 1. Introduction

*Shigella* spp., is very closely related with *Escherichia coli* in terms of phenotypes and genotypes, both of which are Gram-negative bacteria and belong to the *Enterobacterales* phylum (Pizzato et al., 2022). Previously, genetic studies based on multi-locus enzyme electrophoresis (MLEE) at population level identified that *Shigella* spp., fell within *E*. *coli* (Pupo et al., 2000), while pH-indicator-based triple sugar ion (TSI) test showed poor sensitivity and specificity in terms of discriminating *E*. *coli* and *Shigella* spp., from other bacterial species (Rautureau et al., 2019). Other methods commonly used in clinical laboratory such as 16S rRNA gene sequencing and matrix-assisted laser desorption/ionization-time of flight mass spectrometry (MALDI-TOF MS) cannot differentiate *Shigella* spp., from *E*. *coli* (Devanga Ragupathi et al., 2018; van den Beld et al., 2022). Therefore, it is very difficult and time-consuming to separate *Shigella* spp., from *E*. *coli* in clinical laboratories, though the two groups of bacteria show differences in epidemiology and clinical infections (Devanga Ragupathi et al., 2018), and *Shigella* spp., have been confirmed to be not clones of *E*. *coli* but sister species in the genus *Escherichia* (Zuo et al., 2013).

From clinical perspective, *Shigella* spp., are responsible for life-threatening dysentery (also known as shigellosis) and the infection leads to high childhood morbidity and mortality globally (Wang et al., 2019; Zhu et al., 2020), while *E*. *coli* are normally commensals found in human gut microbiota (Rautureau et al., 2019). However, among the many *E*. *coli* sub-strains, enteroinvasive *E*. *coli* (EIEC) behaves similarly as *Shigella* spp., by causing invasion and inflammatory destruction of the human colonic epithelium, which also leads to the development of shigellosis (Belotserkovsky and Sansonetti, 2018). In fact, both EIEC and *Shigella* spp., evolved from commensal *E*. *coli* through loss and/or gain of functional genes and virulence genes (Belotserkovsky and Sansonetti, 2018). Therefore, it is important to rapidly and accurately discriminate *E*. *coli* from *Shigella* spp., in order to facilitate the clinical diagnosis, prevention and control of *Shigell*a spp., and *E*. *coli* infections.

Since both *Shigella* spp., and *E*. *coli* are able to grow on distinctive selective media, traditional biochemical tests and serotyping experiments are regularly used for their discrimination in clinical labs (Pizzato et al., 2022). However, these methods suffer from multi-step complexity and reliance on commercially-available kits, which makes the differentiation procedure time-consuming, sophisticated, and expensive, calling for the development of novel techniques and methods in the field (Pizzato et al., 2022). Recently, as a rapidly developed emerging technique, surface enhanced Raman spectrometry (SERS) has been extensively explored for its potential and promising application in bacterial pathogen detection and antibiotic resistance profiling (Tang et al., 2021; Wang et al., 2021; Liu et al., 2022; Tang et al., 2022). There are currently multiple studies using SERS technique combined with machine learning models to detect different types of bacterial pathogens, which have been summarized in recent reviews and will not be described in details here (Liu et al., 2017; Lee et al., 2021; Wang et al., 2021; Rebrosova et al., 2022). Therefore, SERS technique holds promising future for rapid and non-invasive diagnostics of bacterial pathogens in clinical settings. In addition, Chen et al. (2022) developed nanoporous Ag nanorods as SERS substrates to sensitively detect adenine, spike glycoprotein, and different types of bacteria, while Liu et al. (2022) constructed novel Ag nanoparticles that are able to generate high antibacterial activities.

Although many studies have applied SERS technique in the rapid identification of bacterial pathogens, there are currently few studies focusing on using the combination of SERS technique and machine learning models to discriminate *Shigella* spp., from *E*. *coli*. In this study, we collected SERS spectra for *Shigella* spp., and *E*. *coli via* handheld Raman spectrometer, which were then analyzed *via* machine learning algorithms. According to the results, *Shigella* spp., could be rapidly and accurately discriminated from *E*. *coli* through the combination of SERS technique and machine learning algorithms, which provides a promising future for the application of SERS technique in diarrheal prevention and control in clinical settings.

## 2. Methods and materials

### 2.1. Collection of bacterial strains

Ten *E*. *coli* strains and eight *Shigella* strains were used in this experiment. Two of the laboratory strains *E*. *coli* BL21 (DE3) and BW25113 was purchased from Tiangen Biotech Co. Ltd (Beijing, China), the remaining eight *E*. *coli* strains and eight *Shigella* spp., strains (*S*. *dysenteriae*: 2 strains, *S*. *boydii*: 2 strains, *S*. *flexneri*: 2 strains, and *S*. *sonnei*: 2 strains) were directly isolated from clinical samples and confirmed *via* both biochemical tests and mass spectrometry in Xuzhou Infectious Diseases Hospital affiliated to Xuzhou Medical University. All the strains were stored in liquid Luria Broth (LB) culture with 25% glycerol stock at −80°C. During the study, all the strains were recovered from the −80°C freezer by streaking on LB agar plate and incubating at 37°C overnight before experimental analysis.

### 2.2. Preparation of silver nanoparticles as surface enhanced Raman spectroscopy substrate

The preparation procedures for silver nanoparticles (AgNPs) have been well documents in previous studies (Tang et al., 2021; Liu et al., 2022; Tang et al., 2022; Wang et al., 2022a,b). Therefore, we only briefly stated the preparation steps in terms of the key reactions. In particular, 33.72 mg of silver nitrate (AgNO_3_) was added to a triangular flask with 200 mL of deionized distilled water (ddH_2_O) with stirring and heating until boiling. 8 mL of sodium citrate (Na_3_C_6_H_5_O_7_) was then added with stirring and heating at 650 r/min for 40 min. Stop heating and keep stirring until the solution cools down to room temperature, which was then filled up to 200 mL with ddH_2_O. 1 mL of the prepared solution was transferred to a clean Eppendorf (EP) tube for 7-min centrifugation at 7000 r/min. Discard the supernatant and resuspend the pellet with 100 μL of ddH_2_O, which was the AgNP substrate that were stored without light at room temperature for long-term use.

### 2.3. Surface-enhanced Raman spectroscopy

For each bacterial species (10 *E*. *coli* strains and eight *Shigella* spp., strains) used in this study, after recovering *via* cultivating on LB agar plate overnight, a single colony was selected and inoculated into 15 μL phosphate buffer saline (PBS) and well mixed *via* vortexing, which was then well mixed with 15 μL negatively-charged AgNPs substrate solution. The well-mixed suspension was dropped onto the surface of silicon wafer and to form a circular spot of suitable size, which was dried naturally before SERS spectroscopy. The hand-held Raman spectrometer Anton Paar™ Cora100 (Anton Paar Shanghai Trading Co., Ltd., China.) was used for sample analysis and Raman spectral detection by using the following settings of parameters: (1) excitation wavelength: 784.56 nm; (2) excitation power: 25 mW; (3) spectral resolution: 1 nm; (4) spectral wave number resolution: 10 cm^−1^; (5) detection spectral range: 400–2,300 cm^−1^. All the SERS spectra were calibrated using the Raman peak at 520 cm^−1^ as the reference peak and the dark current was deducted during integration time. For each bacterial species, a total of 200 spectra were collected by randomly selecting detecting sites in each of the dried sample spots. Therefore, a total of 800 spectra for *Shigella* spp., and 800 SERS spectra of *E*. *coli* were used for further analysis in this study,

### 2.4. Average surface enhanced Raman spectroscopy spectra and characteristic peaks

#### 2.4.1. Calculational analysis of average surface enhanced Raman spectroscopy spectra

By calculating the average intensities under each Raman shift for all the SERS spectra that were generated from *E*. *coli* (*N* = 800) and *Shigella* spp (*N* = 800), respectively, average Raman spectra were then obtained. 20% of standard deviation (SD) was also calculated and visualized as shaded region around the average SERS spectra for *E*. *coli* and *Shigella* spp. Average Raman spectra with standard error bands were plotted using Origin software (OriginLab, United States). The width of the error bands showed the reproducibility of Raman spectra. The wider the error band, the worse the reproducibility.

#### 2.4.2. Computational identification of characteristic peaks

Characteristic peaks for the SERS spectra of *E*. *coli* and *Shigella* spp., were conducted *via* the software LabSpec6 (HORIBA Scientific, Japan). In specificity, *GaussLoren* function was used for the analysis with parameters set to Level = 13%, Size = 20, and Iteration = 5, while other parameters were in default. All the identified characteristic peaks were marked with black arrows and labelled with corresponding Raman shifts along the average SERS spectra. Biological meanings of all the characteristic peaks were sourced from literature.

### 2.5. Surface enhanced Raman spectroscopy spectral deconvolution and barcoding

In order to explore the characteristic features of the average Raman spectra for *Shigella* spp., and *E*. *coli*, we performed deconvolution operations on each average Raman spectrum. In specificity, baseline correction was first performed on the average spectrum with the parameter set to type = Polynom and degree = 4, which aims to highlight the representative characteristic peaks so that finer differences can be found by means of deconvolution. The function *fit peaks* pro in Origin (version 2019b 32bit) was applied for peak fitting, which used *Voigt* function maximum value and half width to obtain the Doppler and Lorentz lines to solve the deconvolution sub-bands of spectra. Before fitting, appropriate values for the *Gaussian width* and *Lorentzian width* parameters were selected to avoid fitting failures. In addition, barcodes are an efficient mean for electronic record-keeping, which enables rapid identification *via* software (Pezzotti et al., 2021). Therefore, in this study, in order to improve the accessibility of spectral data, we designed barcodes for the deconvoluted Raman spectra in terms of different variants and their subtypes. The barcodes were generated through assigning each band a line with width equal to 1/30 of the width of the sub-band and a distance from the successive line proportional to the band area, which provided necessary flexibility and rapidity for Raman spectroscopy methods.

### 2.6. Unsupervised clustering analysis of surface enhanced Raman spectroscopy spectra

In order to investigate the inherent differences among SERS spectra of *E*. *coli* and *Shigella* spp., T-Distributed Stochastic Neighbor Embedding (T-SNE) algorithm was used for dimensionality reduction and clustering visualization of bacterial spectral data. Set n_components = 2 and learning_rate = 10 for the TSNE function in the *scikit-learn* package (version 0.21.3) to analyze the spectral difference between *Shigella spp*., and *E*. *coli* spectra. For data visualization, we chose the first two feature dimensions after TSNE dimensionality reduction as the coordinate axises, and displayed the categories of *Shigella* spp., and *E*. *coli* in the form of scatter plots.

### 2.7. Supervised machine learning analysis

#### 2.7.1. Supervised clustering analysis of surface enhanced Raman spectroscopy spectra

Orthogonal partial least squares discriminant analysis (OPLS-DA) algorithm was used in this study to visually distinguish *E*. *coli* and *Shigella* spp., based on their SERS spectra. In specificity, all SERS data were imported into the software SIMCA (Umetrics, Sweden). Select OPLS-DA as the model type, and then click *Autofit* button to fit the model. The software automatically calculated R2X, R2Y and Q2 to measure model performance. Select the *Scatter* option under the *Scores* function to visualize the classification results of the model in the form of scatter plots. Different spectra data were represented by different colors, and dashed circles and labels were used to indicate the corresponding data categories.

#### 2.7.2. Classical machine learning analysis of surface enhanced Raman spectroscopy spectra

To identify the spectral differences between *E*. *coli* and *Shigella* spp., we applied two classical machine learning (ML) algorithms, Random Forest (RF) and Support Vector Machine (SVM), and deep learning algorithms Convolutional Neural Network (CNN) for SERS spectral analysis. Before performing ML analysis, we used the *train_test_split* function to split all SERS spectral data into training, validation and test sets in a ratio of 6:2:2. The test set data was only used to test the prediction performance of the model on unfamiliar data sets. The two machine learning algorithms were implemented by calling the *RandomForestClassifier* and *SVC* function models in the *scikit-learn* package (version 0.21.3). During training the two machine learning models, we used the *GridSearch* function to train and tuning the model parameters in order to achieve the best fitting effect of ML modes (Supplementary Table S1).

#### 2.7.3. Convolutional neural network analysis of surface enhanced Raman spectroscopy spectra

Due to the high dimension of SERS spectral data and the subtle spectral differences between bacterial species, ML algorithms frequently failed to predict spectra correctly. Since deep learning algorithms have feature extraction performance and does not require excessive parameter optimization and feature engineering, raw SERS spectral data could be directly imported into models to obtain better discrimination and prediction results. CNN mainly consists of convolutional Layer, MaxPooling Layer and Fully Connected Layer. The convolutional layers were used to learn the intrinsic feature representation of the spectral data, and a nonlinear activation function *ReLU* was applied to each convolutional layer to enable the network to detect nonlinear features. In this study, the size of the convolution kernel was set to 3*1, and the size of the filters was set to 8, 8 and 16, respectively. The MaxPooling layer can compress the extracted feature information, so that the entire network can extract a wider range of feature information. In this study, a MaxPooling layer was connected after each two convolutional layers, and the pool_size was set to 3. After multiple convolutional and pooling layers, the global information was input into the Fully Connected layer for classification using the *Softmax* function.

### 2.8. Comparative evaluation of machine learning algorithms

To evaluate the capacities of machine learning algorithms on the differentiation of *Shigella* spp., and *E*. *coli*, quantitative metrics were used to measure the performance of these algorithms. Accuracy (ACC), as the most basic evaluation index in spectral signal identification, is often used to describe the correct prediction of the overall result. In this study, to avoid the data imbalance issue caused by the random splitting of data, we used *Precision* (P) and *Recall* (R) to obtain objective model evaluations. *Precision* represents the probability of a sample that is actually positive among all samples that are predicted to be positive, while *Recall* represents the probability of a sample that is predicted to be positive among all samples that are actually positive. During the data analysis process, the *average* parameters of Precision and Recall were set to *Micro* and *Macro*, respectively. As *Precision* and *Recall* are mutually influencing indicators, *F1-Score* as the harmonic average of the two indicators, were introduced to provide a comprehensive evaluation, and the *average* parameter was weighted. In addition, in order to avoid the occurrence of overfitting of the model during training, we used the five-fold cross-validation method with cv. = 5 in the *cross_val_score* function, which divided the training set data into 5 equal-sized subsets and tested the models consecutively. That is, during the training process, a set of data subsets were selected as the validation set, and the remaining 4 sets were used as the training set, and 5 models were trained to correspond to 5 accuracy rates, respectively, while the average accuracy rate was calculated as the final training effect. In addition, ROC curves and confusion matrix were also drawn to measure the performance of the models. In specificity, we evaluated machine learning models by using *roc_curve* to draw the ROC curve. In addition, the area under the curve (AUC) value calculated by the *roc_auc_score* function was also used as a metric to eliminate the imbalance of sample categories. In order to more intuitively observe the performance of the model on the test set data, we visualized the prediction results of the optimal model CNN *via* confusion matrix *via confusi_matrix* function in the form of a 2*2 matrix.

### 2.9. Interpretive analysis of surface enhanced Raman spectroscopy spectra

**Figure 1:**
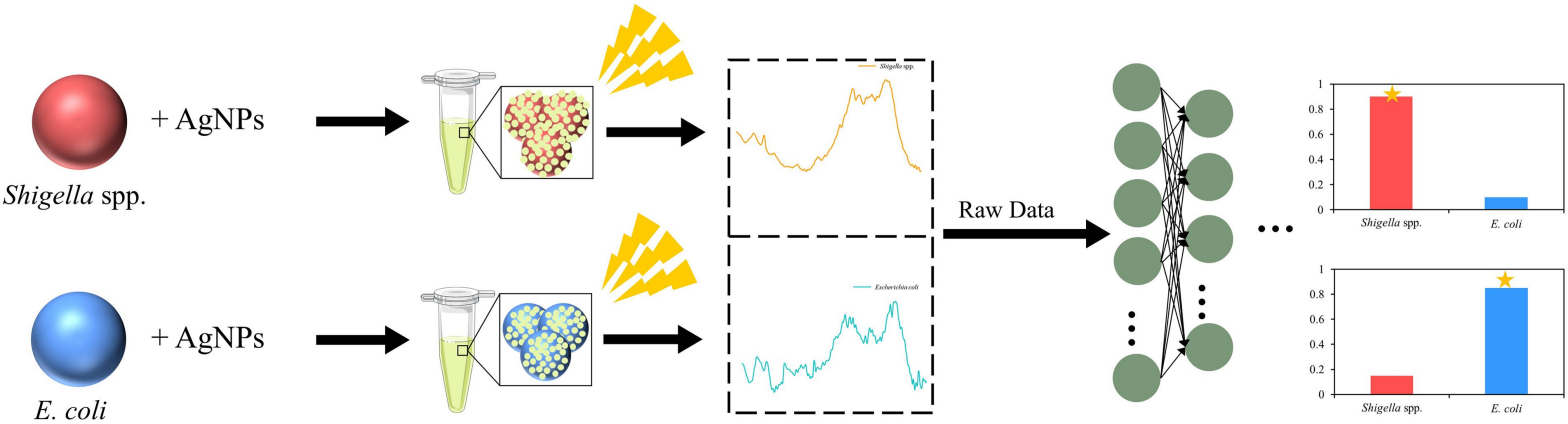
Schematic illustration of bacterial spectral collection and computational analysis of SERS spectra of *Shigella* spp., and *E*. *coli*.

It was worth noting that, in this study, we also used the Grad-CAM algorithm to explain how the deep learning model extracted the SERS spectral features and the decision-making process of the convolutional layer on the SERS spectral signal. In the specific calculation process, the *tf*.*GradientTape* method was conducted to calculate the gradient vector of the last convolutional layer, and the channel mean was calculated using the *tf*.*reduce_mean* method. The Grad-CAM of the target bacterial species was generated according to the gradient vector and the channel mean. In addition to obtaining the weight distribution of the model for the spectral data, we also investigated how the model classified the spectral data of *Shigella* spp., and *E*. *coil*. Therefore, we input the abstract features extracted from the last layer of the convolution layer (num_layer = 5) into TSNE for classification, which further proved the feasibility of the CNN model. During the classification, the TSNE parameters were set to n_components = 2, init = ‘pca’, and random_state = 0. A brief flow chat was given to show the logic of the SERS spectral collection and computational analysis behind the discrimination of *Shigella* spp., and *E*. *coli* (Figure 1).

## 3. Results

### 3.1. Average and deconvoluted surface enhanced Raman spectroscopy spectra

**Figure 2:**
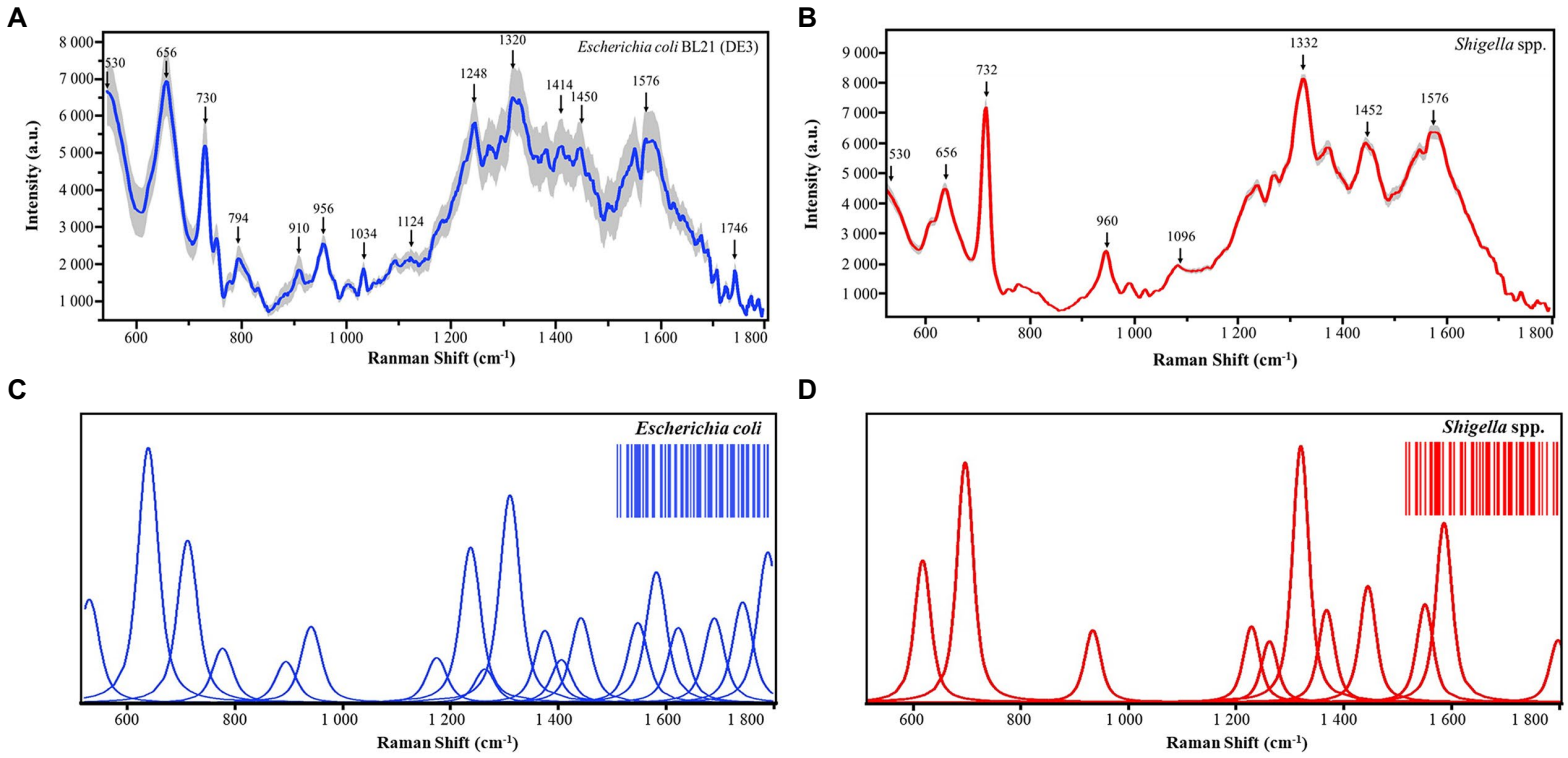
Average and deconvoluted SERS spectra of *Shigella* spp., and *E*. *coli*. **(A,B)** Average SERS spectra of *Escherichia coli* (N = 800) and *Shigella* spp (*N* = 800). **(C,D)** Deconvoluted SERS spectra of *Escherichia coli* and *Shigella* spp., *X*-axis represents Raman shifts in the range of 530–1800 cm^−1^, while *Y*-axis represents the relative Raman intensity. a.u. means artificial unit and has no real meaning. Two-dimensional barcodes at the upper-right corners were generated *via* deconvolution, which were unique for *Escherichia coli* and *Shigella* spp., respectively.

Both bacterial morphological characteristics and physiological states have different molecular basis, while SERS spectra can reflect these molecular differences and are able to transform these chemical and structural information into SERS signal intensities at different Raman shifts (Lu et al., 2020). As for bacterial cells belonging to the same species, they are similar to each other in terms of morphology and physiology, while those from different species are quite dissimilar (Lu et al., 2020). Therefore, different bacterial species could be discriminated based on the analysis of their SERS spectra. In this study, we collected SERS spectra for *Shigella* spp., and *Escherichia coli*, respectively. Average Raman spectra with shaded standard error (SE) bands for the two species were calculated to quantitatively display the general trend of SERS spectral repeats and also reflect the data variance among the spectra (Liu et al., 2022), according to which, SERS spectra were well repeated for *Shigella* spp., and *E*. *coli*, and the reproducibility of SERS spectra varied in acceptable ranges (Figures 2A,B). In this study, deconvoluted SERS spectral band components that were directly linked to molecular structures and chemical components were also generated, which aimed to evaluate SERS spectral characteristics efficiently (Pezzotti et al., 2022). Based on the deconvoluted SERS spectra, two-dimensional (2D) Raman barcoding bands were then generated for *Shigella* spp., and *E*. *coli* at the upper right corners of Figures 2C,D. In particular, the deconvolution spectra consisted of a series of Vogit sub-bands, each representing a spectral characteristic peak. It could be seen that the method fts the important characteristic peaks of Raman spectra well and could eliminate the interference of other artifactual peaks. In addition, *Shigella* spp., and *E*. *coil* Raman barcodes also contained subtle details of the structure of internal metabolites, amplifying the differences among the two bacterial genera. Taken together, the Raman barcodes generated through deconvolution analysis greatly supported the electronic storage of SERS spectra, converted molecular characteristics into information, and improved the recognition of bacterial species.

### 3.2. Characteristic peaks of surface enhanced Raman spectroscopy spectra

As previously mentioned, due to the compositional and physiological differences between bacterial species, SERS spectra contains unique characteristic peaks corresponding to different molecular structures and components (Tang et al., 2021). In this study, under the signal measurement of SERS spectra at the wavelength of 785 nm, characteristic peaks for *E*. *coli* and *Shigella* spp., were computationally identified, respectively. All the characteristic peaks were annotated with a black arrow in Figure 2, while band assignments of characteristic peaks corresponding to potential molecular structure and components were given in details in Table 1. The unique characteristic peaks to both bacteria revealed apparent differences between *E*. *coli* and *shigella* spp. We can see that there are many different peaks between the two bacterial genera. In specificity, as for *E*. *coli*, the unique characteristic peaks identified in its average SERS spectrum include C=O, C-N and ring deformation at 794 cm^−1^ (Kubryk et al., 2016), Ribose vibration, one of the distinct RNA modes at 910 cm^−1^ (Szekeres and Kneipp, 2019), v(C-C) at 1034 cm^−1^ (Athamneh et al., 2014), Amine III of proteins at 1248 cm^−1^ (Bashir et al., 2021), (C-N) stretch at 1320 cm^−1^ (Zeiri et al., 2004), and Stretch C=C in the quinoid ring at 1414 cm^−1^ (Laska and Widlarz, 2005). On the other hand, for *Shigella* spp., their unique characteristic peaks include C-C chain stretch of cell wall lipids at 1096 cm^−1^ (Zheng et al., 2018), tryptophan at 1332 cm^−1^ (Xie et al., 2013), and Ring stretching (Adenine, guanine) at 1594 cm^−1^ (Demirel et al., 2009).

**Table 1:** Band assignments of characteristic peaks to potential metabolites in SERS spectra for *Shigella* spp., and *E*. *coli*.

**Wavenumber (cm** ^**−1**^ **)**	**Band assignment**	*Escherichia* ***coli***	***Shigella* spp.**	**References**
656/658	COO-bending in tyrosine, guanine vibration			Bashir et al. (2021)
730/732	*v*(glycosidic)			Kubryk et al. (2016)
794	C=O, C-N and ring deformation			Kubryk et al. (2016)
910	Ribose vibration, one of the distinct RNA modes			Szekeres and Kneipp (2019)
956/960	δ(C=C)			Xie et al. (2013)
1,034	*v*(C-C)			Athamneh et al. (2014)
1,096	C–C chain stretch of cell wall lipids			Zheng et al. (2018)
1,124	C-C stretching			Lu et al. (2011)
1,248	Amide III (of collagen)			Cheng et al. (2005)
1,320	(C–N) stretch			Zeiri et al. (2004)
1,332	Tryptophan			Xie et al. (2013)
1,414	Stretch C=C in the quinoid ring			Laska and Widlarz (2005)
1450/1452/1454	δ (CH_2_) scissoring			Xie et al. (2013)
1576/1584	*v*(C=C)			Xie et al. (2013)
1,594	Ring stretching (Adenine, guanine)			Demirel et al. (2009)

### 3.3. Clustering analysis of *Shigella* spp., and *Escherichia coli* surface enhanced Raman spectroscopy spectra

**Figure 3:**
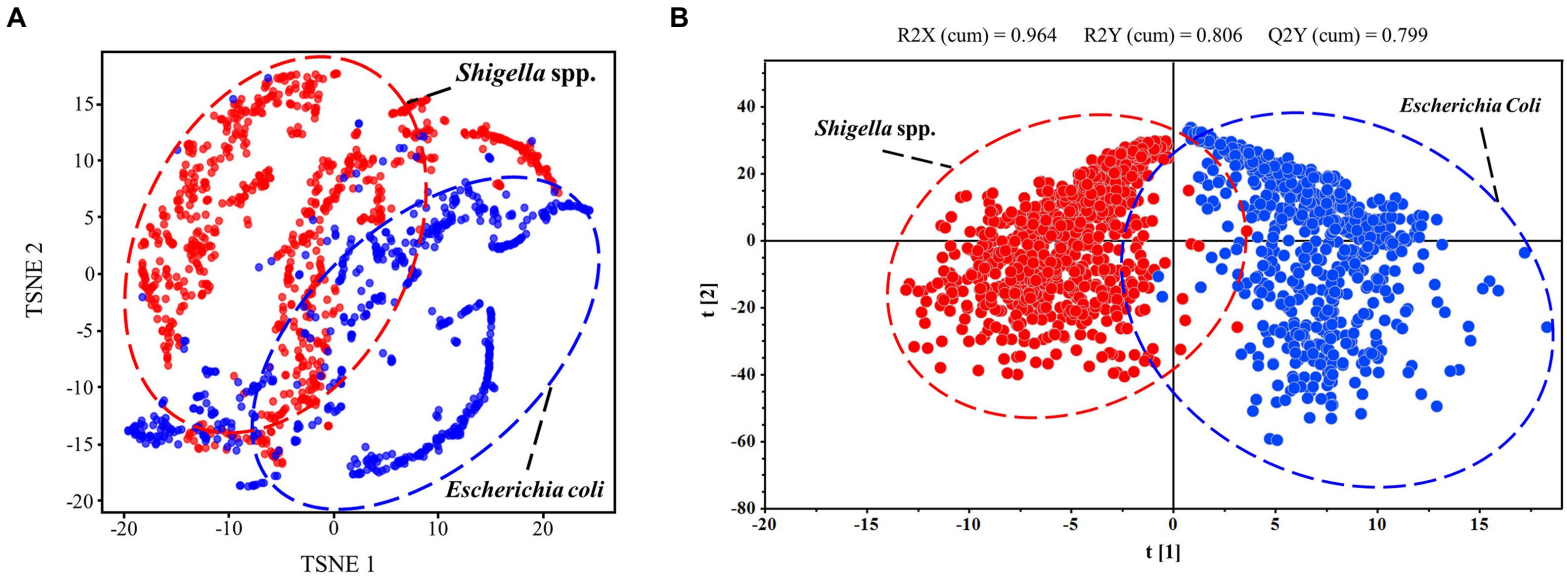
Clustering analysis of SERS spectra of *Shigella* spp., and *E*. *coli* through T-SNE and OPLS-DA algorithms. **(A)** Scatterplot of SERS spectra *via* T-SNE algorithm. **(B)** Scatterplot of SERS spectra *via* OPLS-DA algorithm.

Clustering analysis is able to find commonalities between data elements, therefore dividing SERS spectra into different parts (also known as clusters) so that spectra in the same part are similar to each other while spectra in different parts are dissimilar to each other, leading to the differentiation of bacterial species (Talabis et al., 2015). In this study, we applied the two algorithms, TSNE and OPLS-DA, for the clustering analysis of *Shigella* spp., and *E*. *coli* SERS spectra. TSNE is considered as one of the best high-dimensionality data reduction method and clustering visualization tool, though it has obvious disadvantages of large memory consumption and long running time (Linderman and Steinerberger, 2019; Pezzotti et al., 2019). During the application of TSNE method in SERS spectra, the result showed that two bacterial genera could be well separated into two groups but the classification boundary is not clear due to the mixture of certain number of *E*. *coli* SERS spectra (Figure 3A). As for OPLS-DA, it is a multivariate statistical method for supervised data clustering, which provides insights into the divisions of data groups based on high-dimensional spectral measurements (Worley and Powers, 2016). When using OPLS-DA method for analyzing the same SERS spectral dataset, it was found that *Shigella* spp., and *E*. *coli* spectra were well clustered into two groups with smaller intra-genus variations and the boundary between the two genera was clear (Figure 3B). In addition, unlike TSNE, OPLS-DA gave a quantitative measurement of the clustering result, which showed that R2X (*cum*) = 0.964, R2Y (*cum*) = 0.806 and Q2 (*cum*) = 0.799. The larger the three parameters are, the better the classification performance of the model is, and the difference between R2X and R2Y is generally no more than 0.3 (Abdel-Ilah et al., 2017).

### 3.4. Prediction of *Shigella* spp., and *Escherichia coli* based on surface enhanced Raman spectroscopy spectra

Although clustering analysis could differentiate SERS spectra of *Shigella* spp., and *E*. *coli* into different groups, it is difficult to know the accuracy and robustness of the analysis. Therefore, supervised machine learning analysis was used for predicting the identity of a particular SERS spectra. In particular, supervised machine learning algorithms are able to learn the patterns in the input variable X and the construct mapping functions Y = f(X) in order to achieve the purpose of using unknown input data X to accurately predict its output Y (Tang et al., 2021). In this study, we compared the predictive capacities of three representative machine learning algorithms, CNN, SVM, and RF. The results were shown in Table 2, according to which, all the prediction parameters for the deep learning model CNN reached to values of 99.64%, indicating that CNN model worked the best in capturing the features of the SERS spectral signals and was able to predict *Shigella* spp., and *E*. *coli* with no mistake. As for the two classic machine learning algorithms, it was found that both SVM and RF achieved very high accuracy and robustness, which was very close to the performance of CNN, confirming that SVM and RF were also suitable in predicting SERS spectra of *Shigella* spp., and *E*. *coli*.

**Table 2:** Comparison of the predictive abilities of three supervised machine learning algorithms in the analysis of *Shigella* spp., and *E*. *coli* SERS spectral data.

**Algorithm**	**ACC (%)**	**Recall (%)**	**Precision (%)**	**F1 (%)**	**5-Fold CV (%)**	**AUC (%)**
**CNN**	99.64	99.64	99.59	99.64	99.65	100
**SVM**	98.95	99.31	99.31	99.31	99.21	98.97
**RF**	96.49	96.49	96.11	96.48	97.10	95.69

**Figure 4:**
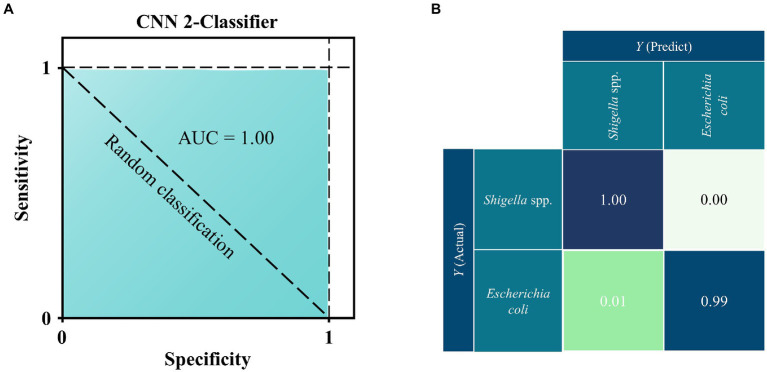
ROC curve and confusion matrix for the deep learning CNN model when applied to the SERS spectra of *Shigella* spp., and *E*. *coil*. **(A)** ROC curve. According to the comparison, CNN achieved the best performance with area under curve (AUC) = 1.00 than all other algorithms. **(B)** Confusion matrix. Numbers in the confusion matrix stood for the percentage of correctly classified (diagonal) or mis-classified (off-diagonal) spectra, respectively.

ROC curve aims to compare the sensitivity and specificity across a range of values for the predictive ability of supervised machine learning methods, and AUC means overall accuracies in distinguishing data samples (Florkowski, 2008). As for confusion matrix, it is a table that shows the classification results based on the true class and predicted class (Tang et al., 2021). Therefore, in this study, we plotted both ROC curve and confusion matrix for the optimal prediction model CNN. As for the ROC curve, the x-axis represents the specificity (false positive rate, FPR) while the y-axis represents the sensitivity (true positive rate, TPR). According to Figure 4A, it could be seen that the AUC value was 1.00, which indicated that the CNN model had very high specificity and sensitivity. The confusion matrix showed the specific performance of the model on the test set data (Figure 4B), It could be seen that the prediction accuracy of CNN models was 100% for *Shigella* spp., and 99% for *E*. *coli*, while 1% of the *E*. *coli* spectra was misidentified as *Shigella* spp., indicating that these spectra could be accurately assigned to the correct species with very low error rate.

### 3.5. Grad-CAM analysis of *Shigella* spp., and *Escherichia coli* surface enhanced Raman spectroscopy spectra

**Figure 5:**
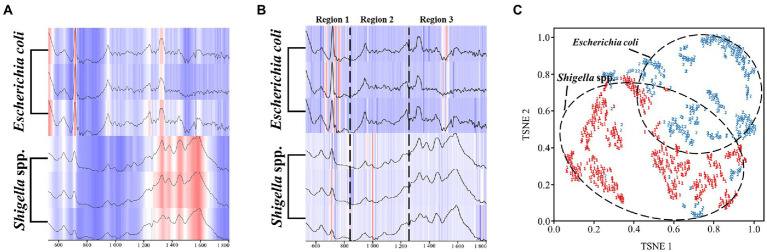
Schematic illustration of the computational mechanisms behind the CNN model classification of *Shigella spp*., and *E*. *coil* SERS spectra. **(A)** Heatmap of raw SERS spectra. **(B)** Model weight allocation in SERS spectra arbitrarily divided into three regions. **(C)** Classification of SERS spectra. For the heatmap, the darker the color, the more attention the CNN model pays. As for the clustering analysis, the two bacterial genera, *Shigella* spp., and *Escherichia coli*, were represented by different labels and colors.

In order to observe the distributions of the CNN model in the SERS spectra of different bacterial genera, we used the Grad-CAM algorithm to analyze the full spectral data. It could be seen that the heatmap distribution of the raw spectra data was determined by the intensity of characteristic peaks, that is, high intensity region appeared in red and low intensity region appeared in white (Figure 5A). As for the spectral data output by the CNN model, they were not classified according to the characteristic peaks as previously inferred. Instead, according to the results, there were obvious differences in the weight distribution between the spectra of the two bacterial genera (Figure 5B). In order to better interpret the result, we arbitrarily divided the heatmap into three regions. In the first region of the spectra, the CNN model assigned more attention in the range of 756-754 cm^−1^ in *E*. *coil*, while more attention was distributed in the range of 688-721 cm^−1^ for the SERS spectra of *Shigella* spp. In the second region, where the spectral intensity was intermediate, the model did not pay much attention to *E*. *coli* in this area, while for *Shigella* spp., the attention of CNN model mainly focused on around the characteristic peak at 994–1000 cm^−1^. In the third region of the spectra, the model focused on the Raman shifts at 1484–1542 cm^−1^ for *E*. *coil*, but for *Shigella*, the model assigned more weights on the end of the spectrum from 1764 cm^−1^ to 1776 cm^−1^. To further demonstrate how the CNN model classified *Shigella* spp., and *E*. *coil*, we used TSNE to show the extraction results of spectral features by the model. It could be seen that the two bacterial groups were divided into two well-separated clusters, and according the label display, it can be judged that the model can distinguish the two bacteria well (Figure 5C). In sum, Grad-CAM analysis confirmed the reliability of the CNN model, indicating that it had the ability to accurately identify *Shigella* spp., from *E*. *coil*.

## 4. Discussion

It is well known that, due to close similarities in both genotypes and phenotypes, it is technically difficult and time-consuming to differentiate *Shigella* spp., from *E*. *coli* (Rautureau et al., 2019), which mainly involves biochemical characteristics and serotyping (Devanga Ragupathi et al., 2018). Recently, van den Beld et al. (2022) reported that MALDI-TOF MS using a customized database, biomarker assignment, or mathematical classifiers cannot differentiate *Shigella* spp., and *E*. *coli*, either, which strengthened the difficulties of the issue. However, as a proof-of-concept, Pizzato et al. (2022) used MALDI-TOF MS with negative ion mode to generate lipid profiles of *Shigella* spp., and *E*. *coli*, through which these species could be discriminated at accurate rates of around 0.9 when paired with in-house machine learning algorithm and top-ranked features. Recently, with the development of whole genome sequencing technology, the advanced method has also been used for the differentiation of *E*. *coli* and *Shigella* spp. According to a study by Chattaway et al. (2017) *Shigella* was successfully identified from *E*. *coli* and accurately differentiated to the species level *via* kmer-based approach. A nuclear magnetic resonance (NMR) based method for the characterizing the metabolomic features of culture media for lactose negative *E*. *coli* and *Shigella* spp., has also been reported recently, according to the results, when coupled with multivariate classification model analysis, it was revealed that the two very closely related species could be separated *via* bacterial metabolic footprints (Rautureau et al., 2019). However, considering the sophisticated procedures and high expenses of these novel methods, it is implausible to adopt the methodologies and apply them to routine laboratory tests.

As an easy-to-learn, low-cost, non-invasive and label-free method, Raman spectroscopy, especially the surface enhanced Raman spectroscopy due to the significantly enhanced Raman signals, has great application potential for rapid and accurate bacterial pathogen identification in clinical settings, though huge challenges exist and the method has been officially used in real-world situation yet (Wang et al., 2021). Previously, the promising SERS technique has been widely used for both genus/species discrimination and antibiotic resistance profiling in clinically important bacterial pathogens with rapid speed and high accuracy (Ho et al., 2019; Tang et al., 2021; Liu et al., 2022; Tang et al., 2022; Wang et al., 2022a,b). However, there is currently no reported of using label-free SERS technique coupled with machine learning algorithms for the differentiation of *Shigella* spp., and *E*. *coli*, which is thoroughly investigated in this study from the comparison of characteristic peaks for unique molecular structure and components, to clustering analysis of distinct spectral groups, and then to species predictions based on supervised machine learning algorithms. According to the results, it was found that the deep learning algorithm CNN had the best performance and could achieve 99.64% accuracy for the discrimination of *Shigella* spp., and *E*. *coli*. The result is consistent with previous studies, in which CNN model has been found to be the best for analyzing and predicting bacterial species (Tang et al., 2021; Liu et al., 2022; Tang et al., 2022; Wang et al., 2022a,b), though other machine learning algorithms such as SVM and RF also achieved very high levels of accuracy for species identification. Therefore, through this study, it was suggested that the label-free SERS technique coupled with machine learning algorithms could be used for the rapid discrimination of *Shigella* spp., and *Escherichia coli*.

## 5. Conclusion and limitations

Rapid and accurate identification and discrimination of *Shigella* spp., and *E*. *coli* is essential in the prevention and control of diarrhea, especially for children under the age of 5 years old. However, current methods suffer from limitations in discriminating the two very closely related bacterial species and novel techniques are urgently needed in clinical laboratories. In this study, we explored the performance of the rapid and sensitive SERS technique and combined it with the advanced deep learning algorithm CNN in order to identify *Shigella* spp., from *E*. *coli* by using the handheld Raman spectrometer. According to the results, the portable spectrometer is sufficient in acquiring high-quality SERS spectra from *Shigella* spp., and *E*. *coli* colonies in clinical lab, which confirms the applicability of the instrument as a POCT device. In addition, SERS spectral analysis revealed that intrinsic differences existed between *Shigella* spp., and *E*. *coli* due to the presence of unique characteristic peaks, which was further confirmed through clustering analysis *via* both unsupervised and supervised machine learning models. As for species prediction, comparative machine learning analysis of SERS spectra showed that the deep learning algorithm CNN had the best performance. However, due to the comparatively small number of *Shigella* spp., and *E*. *coli* strains involved in this study, the capacity of the CNN model generalization might be restricted. In addition, since different bacterial culture media, cultivation conditions, and physiological states could also influence the SERS spectra, more *Shigella* spp., and *E*. *coli* strains cultivated under different conditions should be included for SERS spectral analysis in future investigations, which will greatly improve the robustness and generalization of the constructed machine learning models. Taken together, this study confirmed the application potential of SERS technique *via* handheld Raman spectrometer in the discrimination of *Shigella* spp., and *E*. *coli*, which may facilitate diagnosis, control and prevention of diarrhea in clinical laboratory in near future.

## Data availability statement

The raw data supporting the conclusions of this article will be made available by the authors, without undue reservation.

## Author contributions

LW, XW, and Z-KL conceived and designed the experiments. LW and XW provided platform and resources. LW contributed to project administration and student supervision. LW and WL contributed to the funding acquisitions. WL, J-WT, J-YM, J-WL, Y-WD, Y-LL, and Y-FL carried out the computational and experimental investigations. All the authors wrote and revised the manuscript, and contributed to the article and approved the submitted version.

## Funding

LW was supported by National Natural Science Foundation of China (Grant No. 31900022), Xuzhou Key R&D Plan Social Development Project (Grant No. KC22300), and Jiang-Su Qing-Lan Project (2020).

## Conflict of interest

The authors declare that the research was conducted in the absence of any commercial or financial relationships that could be construed as a potential conflict of interest.

## Publisher’s note

All claims expressed in this article are solely those of the authors and do not necessarily represent those of their affiliated organizations, or those of the publisher, the editors and the reviewers. Any product that may be evaluated in this article, or claim that may be made by its manufacturer, is not guaranteed or endorsed by the publisher.
