# Supplementary material for: Rapid discrimination of Shigella spp. and Escherichia coli via label-free surface enhanced Raman spectroscopy coupled with machine learning algorithms
Source: Front Microbiol. 2023 Mar 8;14:1101357. doi: 10.3389/fmicb.2023.1101357 (PMC10030586; doi:10.3389/fmicb.2023.1101357)
Supplement: Supplementary file 1 [file Table_1.DOCX]

**Supplementary Table S1** Parameter settings for machine learning models optimized for *Shigella* spp. and *E. coil* classification in this study.

| **Models** | **Parameters** | **Values** |
| --- | --- | --- |
| **TSNE** | n_components | 2 |
|  | learning_rate | 60 |
| **RF** | criterion | *entropy* |
|  | max_depth | 3 |
|  | n_estimators | 120 |
| **SVM** | C | 0.01 |
|  | gamma | 0.001 |
|  | kernel | *linear* |
|  | probability | TRUE |
| **CNN** | kernel_size | 3 |
|  | filter | 8, 8, 16 |
|  | pool_size | 3 |
|  | activation | *ReLU*, *Softmax* |
|  | Convolution_Layer | 4 |
|  | Maxpooling_Layer | 2 |
|  | Fullyconnect_Layer | 1 |
